# Two Cases of Surgery

**Published:** 1853-11

**Authors:** Francis Cameron

**Affiliations:** Wentworth, Canada West


### *Two Cases of Surgery.*

BY FRANCIS CAMERON, M. D., WENTWORTH, CANADA WEST.

CASE 1.—*Compound Comminuted Fracture of Humerus.*—An Irish emigrant boy, 15 years of age was thrown from the top of a high load down a bank, causing a compound fracture of the left humerus, just above the condyles. The external condyle was scattered into several pieces, so that I thought it prudent to cut several of the fragments away as the joint was totally disorganized, irritation would have been the consequence of allowing them to remain. A considerable portion of the superior part of the humerus at the fracture being being detached externally, it must be evident that was no easy matter to keep the ends of the fracture in apposition. The laceration at the outside of the joint was fearful, and the whole case seemed to indicate that amputation was the only remedy. This was proposed, but positively objected to by the parents, so that the patient had to take his chance; and as it was in the heat of summer, rendering it impossible to keep worms out of the wound, that chance was a bad one; but through an attention to cleanliness, and keeping him strictly under antiphlogistic regimen, he, to the surprise of every body, got well, with the exception of a rigid joint. What I consider worthy of note in this case is, the lesson it teaches us not to be too precipitate in amputating in such cases, and to be cautious in our prognosis.

CASE 2.—*Penetrating Wound of Chest*—A man about 30 years of age, fell on the sharp end of a harrow tooth, which, passing between the second and third ribs about two inches from the left side of the sternum, penetrated the left lung. Frothy and scarlet colored blood issued from the external wound with a gurgling sound, and in expiration the air would rush out with a hiss. The metal tooth had penetrated about 3 inches, by the appearance of the blood on it, and left an opening through which the lung was visible. I endeavored to heal the wound at first, by the first intention. His friends thought he was doing well as the wound seemed to heal, and only a little air escaped through it. I soon found, however, that the surface of the wound, bruised as it was by so blunt an instrument, must slough before healing could take place; and so made up my mind to let it fill up by granulations from the bottom. I dressed it with common dressings, under which treatment, it soon closed, and combating a strong tendency to inflammation of the wounded lung, I had the pleasure of seeing my patient in a fortnight relieved from danger, and rapidly convalescing. The fact that to my mind seems most worthy of consideration in this case is, that, under certain circumstances, wounds of the lung heal as rapidly as the same class of wounds in other parts of the body.—*Med. Chronicle.*

